# Heteroplasmy in the Mitochondrial Genomes of Human Lice and Ticks Revealed by High Throughput Sequencing

**DOI:** 10.1371/journal.pone.0073329

**Published:** 2013-09-13

**Authors:** Haoyu Xiong, Stephen C. Barker, Thomas D. Burger, Didier Raoult, Renfu Shao

**Affiliations:** 1 Parasitology Section, School of Chemistry and Molecular Biosciences, The University of Queensland, Brisbane, Queensland, Australia; 2 Unité de Recherche sur les Maladies Infectieuses et Tropicales Emergentes (URMITE), UMR CNRS 6236 IRD 198, Faculté de Mé decine, Mé diterranée Infection, Aix-Marseille Université, Marseille, France; 3 GeneCology Research Centre, Faculty of Science, Health, Education and Engineering, University of the Sunshine Coast, Maroochydore, Queensland, Australia; The Centre for Research and Technology, Hellas, Greece

## Abstract

The typical mitochondrial (mt) genomes of bilateral animals consist of 37 genes on a single circular chromosome. The mt genomes of the human body louse, *Pediculus humanus*, and the human head louse, *Pediculus capitis*, however, are extensively fragmented and contain 20 minichromosomes, with one to three genes on each minichromosome. Heteroplasmy, i.e. nucleotide polymorphisms in the mt genome within individuals, has been shown to be significantly higher in the mt *cox1* gene of human lice than in humans and other animals that have the typical mt genomes. To understand whether the extent of heteroplasmy in human lice is associated with mt genome fragmentation, we sequenced the entire coding regions of all of the mt minichromosomes of six human body lice and six human head lice from Ethiopia, China and France with an Illumina HiSeq platform. For comparison, we also sequenced the entire coding regions of the mt genomes of seven species of ticks, which have the typical mitochondrial genome organization of bilateral animals. We found that the level of heteroplasmy varies significantly both among the human lice and among the ticks. The human lice from Ethiopia have significantly higher level of heteroplasmy than those from China and France (P_t_<0.05). The tick, *Amblyomma cajennense*, has significantly higher level of heteroplasmy than other ticks (P_t_<0.05). Our results indicate that heteroplasmy level can be substantially variable within a species and among closely related species, and does not appear to be determined by single factors such as genome fragmentation.

## Introduction

Mitochondrial (mt) genomes exist usually in multiple copies per cell and vary in copy number among different cells and organisms. For instance, there are 30 to 40 copies of mt DNA molecules in the sperm of *Caenorhabditis elegans* whereas ∼250 copies of mt DNA molecules in the oocyte [1]. A mouse sperm has ∼100 copies of mt DNA molecules whereas a mouse oocyte can have up to 100,000 copies of mt DNA molecules [2]. In humans, a sperm has 100 to 1,500 copies of mt DNA molecules whereas an oocyte can have up to 400,000 copies [3], [4], [5], [6]. The multiple copies of mt DNA molecules in a cell or an organism often are not identical in sequence. Heteroplasmy, i.e. variation in mt genome sequence within a cell, an organism or an individual, is common in bilateral animals but usually occurs at very low levels [7], [8], [9], [10], [11].

Heteroplasmy has been most studied in humans in association with mt diseases, aging and cancers; nevertheless, heteroplasmy is present in health individuals too [10], [12], [13], [14]. In arthropods, heteroplasmy in the mitochondrial A+T-rich regions (also called control regions) has been most studied [15], [16], [17]; heteroplasmy in mt tRNA genes and protein-coding genes has also been reported [18], [19], [20]. Andrea *et al*. (1992) showed deletion heteroplasmies in six protein-coding genes and four tRNA genes in *Drosophila subobscura* [19]. Leeuwen *et al*. (2008) showed that heteroplasmy in *cytb* gene was linked to resistance to insecticide bifenazate in mites; such resistance was inherited in a non-Mendelian manner [20].

For bilateral animals, the mt genomes typically consist of a single circular chromosome that is ∼16 kb in size and has 37 genes [4], [21]. The mt genomes of the human body louse, *Pediculus humanus*, and the human head louse, *Pediculus capitis*, however, contain 20 minichromosomes; each minichromosome is 3–4 kb in size and has one to three genes [22], [23]. Herd *et al*. (2012) showed that the heteroplasmy level in the mt *cox1* gene of the human body lice and the human head lice is much higher than that in humans and other animals [24]. At a lower coverage (∼5x) with Sanger sequencing, Herd *et al*. (2012) found 13 heteroplasmic sites in *cox1* gene in human body lice and four heteroplasmic sites in this gene in human head lice, in comparison to only one heteroplasmic site in this gene in humans revealed by high throughput sequencing [9]. Herd *et al*. (2012) suggested that the high-level heteroplasmy might be linked to the fragmented mt genome organization in the human lice, possibly through increased recombination activities between minichromosomes. In the present study, we investigated further the link between heteroplasmy level and mt genome organization by sequencing in high coverage the entire coding regions (15.4 kb) of all of the mt minichromosomes of six human body lice and six human head lice collected in Ethiopia, China and France with an Illumina HiSeq platform. For comparison, we also sequenced the entire coding regions of the mt genomes of seven species of ticks. Ticks are arthropods, so are human lice. Ticks, however, have the typical mt genome organization of bilateral animals [25], [26], [27], [28], [29]. Comparison of human lice with ticks, thus, will allow us to test whether or not heteroplasmy level is indeed linked to changes in mt genome organization.

## Materials and Methods

### Ethical statement

The research ethics committees of The University of Queensland and the University of the Sunshine Coast were consulted for the experimental work undertaken. No ethical approval or permits were required for conducting the research reported in this manuscript according the laws and regulations in Australia and Queensland.

### Sample collection, DNA extraction, PCR amplification and Illumina sequencing

Total DNA was extracted individually from six human body lice, *Pediculus humanus*, six human head lice, *Pediculus capitis*, from Ethiopia, China and France, and seven species of ticks with DNeasy Tissue Kit (QIAGEN) (Table 1). For the human lice, a forward primer (USF1PH1: 5′-GAAATTAAAATTTCAACAAATCTCAACTCG-3′) and a reverse primer (PHR10: 5′-CCCCCCAAGCTATTTATAGCTTGGAGTATTAACGG-3′) were designed from two conserved sequence blocks adjacent to the coding region of each mt minichromosome of the human body louse and the human head louse [19], [23]. These primers were used to amplify the coding regions of the 20 mt minichromosomes of the human body lice and the human head lice. The PCR conditions were: 96°C for 2 minutes; 39 cycles of 98°C for 10 seconds, 62°C for 30 seconds, 72°C for 2.5 minutes; followed by 72°C for 5 minutes and then 25°C for 2 minutes. Each 25-µl PCR reaction contains 0.5 µl La Taq, 2 µl forward primer (10 µM), 2 µl reverse primer (10 µM), 4 µl dNTP (2.5 mM), 2.5 µl La Taq Buffer (10x), 1 µl DNA template, and 13 µl deionized water. Negative controls were always included for contamination check. PCR amplicons were purified and concentrated with the Wizard® SV Gel and PCR Clean-Up System (Promega) and then submitted to the Beijing Genome Institute (BGI) for deep sequencing. Sequencing libraries were constructed for each louse and tick sample at the BGI. For library construction, PCR amplicons were sheared into 180-bp fragments, purified and then end-repaired. Adapters were ligated to both ends of sheared fragments; DNA cluster preparation were then performed and the products were sequenced with an Illumina Hiseq 2000 platform at the BGI (details available at: http://bgitechsolutions.com/service-solutions/services/genomics/de-novo-sequencing; http://support.illumina.com/sequencing/documentation.ilmn).

**Table 1:** Samples of human lice and ticks used in this study.

Sample	Country	Host
Body louse, *Pediculus humanus* (B2470B1)	China	Human
Head louse, *Pediculus capitis* (B2471H5)	China	Human
Body louse, *Pediculus humanus* (B2557B)	France	Human
Head louse, *Pediculus capitis* (B2557H)	France	Human
Body louse, *Pediculus humanus* (B2558B)	France	Human
Head louse, *Pediculus capitis* (B2558H)	France	Human
Body louse, *Pediculus humanus* (B2516B2)	Ethiopia	Human
Head louse, *Pediculus capitis* (B2516H2)	Ethiopia	Human
Body louse, *Pediculus humanus* (B2560B)	Ethiopia	Human
Head louse, *Pediculus capitis* (B2560H)	Ethiopia	Human
Body louse, *Pediculus humanus* (B2563B2)	Ethiopia	Human
Head louse, *Pediculus capitis* (B2563H)	Ethiopia	Human
Hard tick, *Haemaphysalis formosensis* (B2340)	Japan	Unknown
Hard tick, *Haemaphysalis parva* (B2345)	Romania	Unknown
Hard tick, *Rhipicephalus microplus* (B2487)	Cambodia	Cattle
Hard tick, *Amblyomma cajennense* (B2479)	Brazil	Monkey
Hard tick, *Rhipicephalus geigyi* (B2538)	Burkina Faso	Unknown
Soft tick, *Argas* sp. (B2384)	Springbok	Bird
Soft tick, *Otobius megnini* (B2512)	Madagascar	Horse

For the tick species, short regions of *cox1, cytb* and *rrnS* genes were amplified first and sequenced using Sanger method with arthropod-conserved primers [30], [31]. Specific primers were then designed for each species from these regions, and used in most species with conserved tick primers in *cytb* and *rrnS*, designed from known sequences (Table S1). Entire mitochondrial genomes were then amplified in two overlapping fragments for the soft ticks: one fragment from *cytb* to *cox1*, and the other from *cox1* to *cytb*. The mt genomes of hard ticks were amplified in three overlapping fragments that span from *cytb* to *c*o*x1*, from *cox1* to *rrnS*, and from *rrnS* to *cytb*. The long amplicons from the soft and hard ticks were subject to Illumina sequencing. TaKaRa Ex Taq DNA polymerase kit was used to amplify the short *cox1*, *cytb* and *rrnS* fragments (<1 kb). Expand Long Range dNTPack kit (Roche) was used to amplify the long fragments (>1 kb). PCR conditions were optimized for each reaction, with the annealing temperature adjusted to suit the primers used, and the extension time set to one minute per kb of expected amplicon. General PCR conditions for Ex Taq were: 94°C for 60 seconds, followed by 40 cycles of 98°C for 10 seconds, 60°C for 30 seconds, 72°C for 1 minute and a final extension of 72°C for 2 minutes. General PCR conditions for Expand dNTPack were: 92°C for 2 minutes, 10 cycles of 92°C for 10 seconds, 55°C for 15 seconds, 60°C for 8 minutes, followed by 25 cycles of 92°C for 10 seconds 55°C for 15 seconds, 60°C for 8 minutes (increasing by 20 seconds per cycle), and a final extension of 68°C for 7 minutes. PCR products were examined on 1% agarose gel stained with ethidium bromide. DNA Molecular Weight Marker VII (Roche Diagnostics) and Low DNA Mass Ladder (Invitrogen) were used, respectively, to estimate the length and concentration of PCR products. Wizard SV Gel and PCR Clean-up System (Promega) was used to purify PCR products for sequencing. Sanger sequencing was with the ABI Prism BigDye v3.1 Terminator kit (Applied Biosystems) and the Applied Biosystems 3730*xl* DNA Analyzer at the Australian Genome Research Facility (AGRF). Illumina sequencing was with the HiSeq 2000 platform at the BGI, as stated above for the human lice. The sequence reads from the mitochondrial genomes of the human lice and ticks have been deposited in NCBI Sequence Read Archive (SRA) under the accession number SRR616254 and SRR616964.

### Sequence and statistical analyses

Raw Illumina sequence-reads were processed before assembly. The adapter sequences were removed from each sequence read; the reads that contain more than 50% low quality bases (quality value < = 5) were excluded from further analysis. The Q20 bases rate of each pool is above 99.26% (Q20 refers to less than 1% error rate in the sequencing base reads). Processed sequence reads were assembled into contigs with Geneious (Biomatters), using the mitochondrial genome sequences of the human lice and ticks available as references [22], [23], [29]. The assembly parameters were: minimum overlap length 80 bp, and minimum identity 95%. Consensus sequences were made for each contig with the “Generate consensus sequence” function in Geneious with the settings: 1) Threshold: Highest Quality – use chromatogram quality to call the best base; and 2) Assign Quality: Total – Sum quality of contributing bases minus non-contributing bases. Protein-coding genes and rRNA genes were identified with BLAST searches of GenBank [32], [33]. tRNA genes were identified with tRNA-Scan [34] and ARWEN [35]. Heteroplasmy was measured with the “Find SNPs/variations” function in Geneious under the threshold “minimum variant frequency” >1.5%, and “find SNPs only”.

Statistic analyses were conducted to estimate the false positive rate and the false discovery rate for the heteroplasmy called above, according to Storey and Tibshirani (2003) [36]. The false positive rate, indicated as p-value in Storey and Tibshirani (2003), was measured as the probability of a heteroplasmy to be false positive (P_f_) under a binomial distribution given the sequence-read coverage of that heteroplasmic site and the sequencing error of the Illumina platform. P_f_ was calculated with the formula P_f_  = C (n,k) * p∧k * (1-p)∧(n-k), where n is the sequence-read coverage of a heteroplasmy called at a given site of a contig; k is the times of that heteroplasmy seen at that site; C(n, k) is n!/(k! * (n-k)!); and p is the sequencing error, 0.5%, of the Illumina platform. The false discovery rate, indicated as q-value in Storey and Tibshirani (2003), was measured as the probability of a statistically significant heteroplasmy to be false positive (Q_f_). Q_f_ was calculated from the P_f_ values obtained above with the QValue program [36], [37]. The statistical significance threshold for the heteroplasmy called was set at P_f_<1% and Q_f_<0.1%. Measurement of heteroplasmy was further corrected by excluding those at the sequencing-error hotspots revealed in Nakamura *et al*. (2011) and Minoche *et al*. (2011): 1) after four or more identical base calls; and 2) two adjacent SNP variants [38], [39]. One sample T test and Independent samples T test (P_t_) were used to compare the mean heteroplasmy levels between any two samples or genes.

## Results

### Heteroplasmy in the human lice

**Figure 1:**
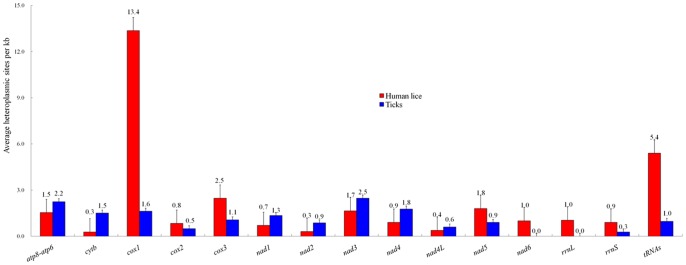
Level of heteroplasmy in the mitochondrial genes of human lice and ticks. Error bars stand for the standard error of the mean value. *Amblyomma cajennense* was excluded due to its extremely high level of heteroplasmy relative to other ticks.

**Figure 2:**
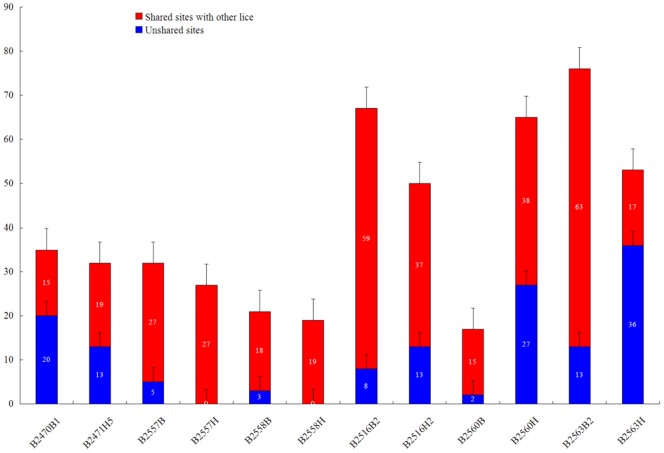
Numbers of shared and unshared heteroplasmic sites in human lice. Error bars stand for the standard error of the mean value.

Twenty contigs were obtained for each of the six human body lice and six human head lice, corresponding to the 20 mt minichromosomes of these lice. The gene content and gene arrangement in each minichromosome of these lice are the same as that reported in Shao et al. (2009, 2012) [22], [23]. At the “minimum variant frequency” >1.5%, P_f_<1% and Q_f_<0.1%, a total of 494 heteroplasmic sites (excluding those at sequencing-error hotspots here and throughout this manuscript) were found in the mt coding regions (15.4 kb) of six human body lice and six human head lice, i.e. an average of 41.1±20.3 (mean ± standard deviation, n = 12) heteroplasmic sites per louse (Table 2). These heteroplasmic sites have an average coverage of ∼6,000x. Of the 494 heteroplasmic sites, 374 sites are in the protein-coding genes, 21 heteroplasmic sites in the rRNA genes, and 99 heteroplasmic sites in the tRNA genes (Table 2, Table S2). Of the 374 heteroplasmic sites in the protein-coding genes, 252 sites are synonymous, i.e. resulting in no amino acid changes, whereas the other 122 sites are nonsynonymous, i.e. resulting in amino acid changes (Table 2, Table S6). There is no significant variation in the level of heteroplasmy among different mt genes in the human lice with the exception of *cox1* gene, which has significantly higher level of heteroplasmy (13.3±15.7 sites per kb, n = 12) than other genes (P_t_<0.05) (Figure 1; Table 3). Each of the 12 human lice share 15 to 63 heteroplasmic sites with one or more other lice (Figure 2; Table 4; Table S3), indicating part of the heteroplasmic sites are inheritable and have limited conservation within species.

**Table 2:** Heteroplasmy in the mitochondrial coding regions of human lice and ticks.

Number of heteroplasmic sites	Total of 12 lice	Average per louse	Total of seven ticks	Average per tick
All mitochondrial genes	494	41.1	261	37.3
Protein-coding genes	374	31.2	221	31.6
Synonymous	252	21.0	158	22.6
Codon 1^st^	14	1.2	10	1.4
Codon 2^nd^	6	0.5	0	0
Codon 3^rd^	232	19.3	148	21.2
Nonsynonymous	122*	10.1	63	9.0
Codon 1^st^	60	5.0	26	3.7
Codon 2^nd^	54	4.5	24	3.4
Codon 3^rd^	8	0.6	13	1.9
rRNA genes	21	1.8	24	3.4
tRNA genes	99	8.3	16	2.3
Anti-codon 3^rd^	4	0.3	0	0

Note: Heteroplasmy was called at minimum variant frequency >1.5%, false positive rate P_f_<1% and false discovery rate Q_f_<0.1%, excluding those at sequencing error hotpots. There are 13 protein coding genes (*atp8-atp6*, *cytb*, *cox1-3*, *nad1-6* and *4L*), two rRNA genes (*rrnL* and r*rnS*), 22 tRNA genes. *46 of the 122 sites have amino acid hydrophobic or hydrophilic changes; 25 of the 122 sites have amino acid polar or non-polar changes.

**Table 3:** Number of heteroplasmic sites in the mitochondrial genes of the human lice.

Human lice	*atp8-atp6*	*cytb*	*cox1*	*cox3*	*nad1*	*nad5*	*nad6*	*nad4L*	*nad4*	*nad2*	*nad3*	*cox2*	*rrnL*	*rrnS*	*tRNAs*
B2470B1	1(1)	3(2)	15(9)	4(3)	1(1)	2(2)	0(0)	0(0)	3(2)	0(0)	0(0)	1(0)	1	0	4
B2471H5	2(2)	0(0)	8(2)	0(0)	0(0)	7(4)	1(1)	0(0)	1(1)	0(0)	2(0)	1(1)	0	0	10
B2557B	2(2)	0(0)	2(2)	3(2)	0(0)	5(3)	0(0)	1(0)	0(0)	0(0)	1(0)	1(1)	2	1	14
B2557H	0(0)	0(0)	2(1)	3(2)	0(0)	5(3)	1(1)	0(0)	1(1)	0(0)	0(0)	0(0)	3	3	9
B2558B	1(1)	1(1)	1(1)	3(2)	0(0)	2(1)	0(0)	0(0)	0(0)	0(0)	0(0)	0(0)	2	2	9
B2558H	1(1)	0(0)	0(0)	3(3)	0(0)	5(2)	0(0)	0(0)	0(0)	0(0)	0(0)	0(0)	0	0	10
B2516B2	0(0)	0(0)	52(4)	0(0)	0(0)	3(3)	1(1)	0(0)	1(1)	0(0)	0(0)	1(1)	2	1	6
B2516H2	0(0)	0(0)	45(1)	2(2)	0(0)	0(0)	0(0)	0(0)	0(0)	0(0)	0(0)	0(0)	0	0	3
B2560B	0(0)	0(0)	4(1)	0(0)	0(0)	3(3)	1(1)	0(0)	0(0)	0(0)	0(0)	1(1)	2	0	6
B2560H	0(0)	0(0)	54(9)	0(0)	0(0)	0(0)	0(0)	0(0)	0(0)	0(0)	2(0)	0(0)	0	1	8
B2563B2	1(1)	0(0)	61(8)	0(0)	0(0)	4(3)	1(1)	0(0)	0(0)	0(0)	1(0)	1(1)	1	0	6
B2563H	7(3)	0(0)	5(2)	6(4)	6(3)	0(0)	1(1)	0(0)	8(4)	4(2)	1(0)	1(1)	0	0	14
Average	1.3	0.3	20.8	2.0	0.6	3.0	0.5	0.1	1.2	0.3	0.6	0.6	1.1	0.7	8.3
A/L	1.54	0.28	13.36	2.47	0.71	1.81	1.00	0.39	0.91	0.31	1.65	0.84	1.05	0.91	5.41

Note: Heteroplasmy was called at minimum variant frequency >1.5%, false positive rate P_f_<1% and false discovery rate Q_f_<0.1%, excluding those at sequencing error hotpots. “H” is for human head lice and “B” is for human body lice. Numbers in bracket refers to the number of nonsynonymous sites of heteroplasmy that result in amino acid changes. A/L stands for Average/Length (kb).

**Table 4:** Number of heteroplasmic sites in the mitochondrial coding regions shared between human body lice and head lice.

	B2471H5	B2557B	B2557H	B2558B	B2558H	B2516B2	B2516H2	B2560B	B2560H	B2563B2	B2563H
B2470B1	0	0	0	0	2	5	1	0	0	1	6
B2471H5		0	2	1	3	5	2	0	0	2	6
B2557B			3	0	5	3	0	1	0	2	11
B2557H				3	5	4	0	1	0	3	7
B2558B					2	2	0	0	0	1	10
B2558H						3	0	0	0	2	9
B2516B2							13	11	14	55	0
B2516H2								0	21	1	1
B2560B									0	0	2
B2560H										1	1
B2563B2											2

The three body lice and three head lice from Ethiopia have an average of 62.2±10.6 heteroplasmic sites per louse (n = 6), which is significantly higher than that of the lice from China (an average of 33.5±2.1 sites per louse, n = 2) (P_t_<0.05) and France (an average of 24.8±5.9 sites per louse, n = 4) (P_t_<0.05) (Table 5). Four heteroplasmic sites in four tRNA genes, *trnN*, *trnD*, *trnT* and *trnM*, are present only in the lice from Ethiopia (n = 6) but not in those from China (n = 2) and France (n = 4) (Table S2). There is no significant difference in the heteroplasmy level among the lice from Ethiopia, nor among those from China and France (P_t_>0.05). There is no significant difference either between the lice from China and those from France in the level of heteroplasmy (P_t_>0.05).

**Table 5:** Heteroplasmy in the mitochondrial coding regions of human lice from China, France and Ethiopia.

Louse sample#	Country	Total heteroplasmic sites	Protein-coding genes	rRNA genes	tRNA genes
B2470B1	China	35	30 (20)	1	4
B2471H5	China	32	22 (11)	0	10
B2557B	France	32	15 (10)	3	14
B2557H	France	27	12 (8)	6	9
B2558B	France	21	8 (6)	4	9
B2558H	France	19	9 (6)	0	10
B2516B2	Ethiopia	67	58 (10)	3	6
B2516H2	Ethiopia	50	47 (3)	0	3
B2560B	Ethiopia	17	9 (6)	2	6
B2560H	Ethiopia	65	56 (9)	1	8
B2563B2	Ethiopia	76	69 (13)	1	6
B2563H	Ethiopia	53	39 (20)	0	14

Note: Heteroplasmy was called at minimum variant frequency >1.5%, false positive rate P_f_<1% and false discovery rate Q_f_<0.1%, excluding those at sequencing error hotpots. “H” is for human head lice and “B” is for human body lice. Numbers in bracket refers to the number of nonsynonymous sites of heteroplasmy that result in amino acid changes.

### Heteroplasmy in the ticks

A single contig was obtained for each of the seven species of ticks, corresponding to the mt chromosomes of these ticks. The mt genomic organization of these ticks is the same as that reported for hard ticks and soft ticks in a number of previous studies [27], [28], [29]. At the “minimum variant frequency” >1.5%, P_f_<1% and Q_f_<0.1%, a total of 261 heteroplasmic sites were found in the seven tick samples, an average of 37.3±22.0 sites per tick (n = 7) (Table 2). These heteroplasmic sites have an average coverage of ∼1,700x. Of the 261 heteroplasmic sites, 221 sites are in the protein-coding genes, 24 sites are in the rRNA genes, and the other 16 sites are in the tRNA genes (Table 2; Table S4). There is no significant variation in the level of heteroplasmy among different mt genes in the ticks (P_t_>0.05) (Figure 1; Table 6). Of the 221 heteroplasmic sites in the protein-coding genes, 63 sites are synonymous whereas the rest are nonsynonymous. No heteroplasmic sites were shared between any two species of ticks except one in *nad3* shared between *Amblyomma cajennense* and *Otobius megnini*, indicating nearly no conservation of heteroplasmy between species. The level of heteroplasmy varies among different species of ticks (Table 7). *Amblyomma cajennense* has 166 heteroplasmic sites (11.5 sites per kb, n = 1) and is significantly higher in heteroplasmy level than the other six ticks (1.1±0.9 sites per tick per kb, n = 6) (P_t_<0.01), and even higher than the human lice (2.9±1.4 sites per louse per kb, n = 12; P_t_<0.01).

**Table 6:** Number of heteropalsmic sites in the mitochondrial genes of ticks.

Ticks	*atp8-atp6*	*cytb*	*cox1*	*cox2*	*cox3*	*nad1*	*nad2*	*nad3*	*nad4*	*nad4L*	*nad5*	*nad6*	*rrnL*	*rrnS*	*tRNAs*
*Haemaphysalis formosensis*	1(1)	5(0)	0(0)	0(0)	0(0)	0(0)	0(0)	0(0)	0(0)	0(0)	0(0)	0(0)	0	0	0
*Haemaphysalis parva*	2(1)	1(1)	0(0)	0(0)	0(0)	2(2)	0(0)	0(0)	0(0)	0(0)	0(0)	0(0)	0	0	1
*Rhipicephalus microplus*	2(1)	3(0)	6(0)	2(0)	3(0)	3(0)	2(0)	0(0)	6(2)	1(0)	6(0)	0(0)	0	0	6
*Amblyomma cajennense*	13(4)	11(10)	22(0)	8(0)	7(3)	13(1)	6(3)	6(1)	19(1)	3(0)	27(10)	1(0)	15	7	8
*Argas* sp.	1(0)	0(0)	2(0)	0(0)	1(0)	0(0)	0(0)	0(0)	3(0)	0(0)	3(0)	0(0)	0	1	0
*Rhipicephalus geigyi*	0(0)	0(0)	1(0)	0(0)	0(0)	0(0)	1(1)	0(0)	5(4)	0(0)	0(0)	0(0)	0	0	1
*Otobius megnini*	5(5)	1(1)	6(0)	0(0)	1(0)	3(3)	3(3)	5(5)	0(0)	0(0)	0(0)	0(0)	0	1	0
Average	3.4	3.0	5.3	1.4	1.7	3.0	1.6	1.6	4.7	0.6	5.1	0.1	2.1	1.3	2.3
A/L	4.16	2.72	3.44	2.08	2.19	3.03	1.68	4.75	3.56	2.15	3.08	0.23	2.19	1.08	1.67

Notes: Heteroplasmy was called at minimum variant frequency >1.5%, false positive rate P_f_<1% and false discovery rate Q_f_<0.1%, excluding those at sequencing error hotpots. Numbers in bracket refers to nonsynonymous sites of heteroplasmy that cause amino acid changes.

**Table 7:** Heteroplasmy in the mitochondrial coding regions of ticks.

Ticks	Total heteroplasmic sites	Protein-coding genes	rRNA genes	tRNA genes
*Haemaphysalis formosensis*	6	6(1)	0	0
*Haemaphysalis parva*	6	5(4)	0	1
*Rhipicephalus microplus*	40	34(3)	0	6
*Amblyomma cajennense*	166	136(33)	22	8
*Argas* sp.	11	10(0)	1	0
*Rhipicephalus geigyi*	8	7(5)	0	1
*Otobius megnini*	24	23(17)	1	0

Note: Heteroplasmy was called at minimum variant frequency >1.5%, false positive rate P_f_<1% and false discovery rate Q_f_<0.1%, excluding those at sequencing error hotpots. Numbers in bracket refers to the number of nonsynonymous sites of heteroplasmy that result in amino acid changes.

## Discussion

Heteroplasmy, i.e. the presence of more than one type of mitochondrial DNA sequence within an individual, is common in humans, other bilateral animals and plants [7]. Heteroplasmy occurs at a relatively low level in humans, with 32 heteroplasmic sites revealed in a human individual by high throughput sequencing at 16,700x coverage [9]. Studies of other animals and plants with low-coverage sequencing also showed low levels of heteroplasmy [12], [18], [20], [40], [41]. Thus, low level of heteroplasmy has been assumed to be a general feature for eukaryotes.

Herd *et al*. (2012) identified, however, substantially more heteroplasmic sites in mt *cox1* gene in the human lice than in humans, with 13 heteroplasmic sites in this gene in human body lice and four heteroplasmic sites in human head lice, in comparison to one heteroplasmic site in this gene in humans revealed by high throughput sequencing [9]. Herd *et al*. (2012) suggested that the high level of heteroplasmy in the human lice might be linked to the fragmentation of mt genomes in these lice, possibly through increased recombination activities between minichromosomes [22], [23]. In the present study, we investigated further whether or not changes in mt genome organization is associated with the level of heteroplasmy by sequencing the entire mt coding region (13 protein-coding genes, two rRNA genes and 22 tRNA genes) of six human body lice, six head lice and seven species of ticks with an Illumina HiSeq platform.

To ensure the accuracy of the measurement of the heteroplasmy level from high throughput sequencing data, we set the minimum heteroplasmy-detection threshold to 1.5%, i.e. only the heteroplasmic sites that have a frequency above 1.5% are counted; sites with a frequency below 1.5% are discarded to exclude sequencing errors. As a general guideline, the sequencing-error rate of the Illumina HiSeq platform is ∼0.5% or less [9], [42], [43]. Our threshold-setting to 1.5% is consistent with those in He *et al*. (2010) and Doan *et al*. (2012), which set the thresholds to 1.6% and 1.5% respectively (i.e. triple the sequencing-error rate) [9], [44]. Furthermore, we set the statistical significance threshold at P_f_<1.0% and Q_f_<0.1%, i.e. the probability of a heteroplasmy we called to be false positive is smaller than 1.0%, and the probability of a statistically significant heteroplasmy we called to be false positive is smaller than 0.1% (Table S5). We also excluded the heteroplasmy that fall into sequencing-error hotspots for Ilumina Hiseq platform revealed by Nakamura *et al*. (2011) and Minoche *et al*. (2011) [38], [39]. By all these measures, we were able to catch the full spectrum of the heteroplasmy while minimize the influence of high throughput sequencing errors.

**Figure 3:**
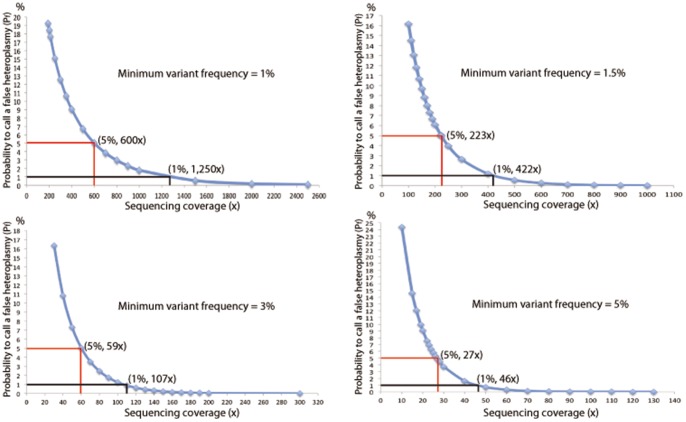
Plots of false positive rate (P_f_) against heteroplasmic-site coverage at minimum variant frequency 1.0%, 1.5%, 3.0% and 5.0% respectively.

We should point out that the parameters we used above to call heteroplasmy from high throughput sequence data, i.e. minimum detection threshold >1.5%, P_f_<1.0% and Q_f_<0.1%, are arbitrary. Indeed, both more relaxed and more stringent parameters can be used, which may increase or decrease the total number of heteroplasmic sites called from the data. The choice of these parameters, however, will not affect the conclusion for the comparison of the heteroplasmy levels between any two samples as long as the same set of parameters are applied. For instance, we also tested minimum detection threshold >1.0%, >3.0% and >5.0%. These three parameters changed substantially the cut-off sequence-read coverage at which heteroplasmy is called (Figure 3) but did not change the conclusion made when minimum detection threshold >1.5% was used.

Despite all having fragmented mt genomes, the human lice from Ethiopia have significantly higher level of heteroplasmy (62.2±10.6 per louse, n = 6) than those from China (33.5±2.1 sites per louse, n = 2; P_t_<0.05) and France (24.8±5.9 sites per louse, n = 4; P_t_<0.05), indicating substantial variation in heteroplasmy level among different populations of human lice. The same is true for the seven species of ticks. Although all of these ticks have the typical mt genomes of animals with all genes on a single chromosome, *Amblyomma cajennense* has significantly more heteroplasmic sites (166, n = 1) than the other six species of ticks (15.8±13.6 sites per tick, n = 1; P_t_<0.05) (Table 7). Fragmentation of mt genome in the human lice does not appear to have any bearing on the level of heteroplasmy as the two human lice from China have similar level of heteroplasmy (33.5±2.1 per louse) as humans, 32 heteroplasmic sites reported in He *et al*. (2010) [9] and 34 sites in Li *et al*. (2012) [45], whereas the four human lice from France have slightly lower level of heteroplasmy (24.8±5.9 sites per louse) than humans (P_t_>0.05). On the other hand, the tick, *Amblyomma cajennense*, has significantly higher level of heteroplasmy (166, n = 1) even than the human lice from Ethiopia (62.2±10.6 per louse, n = 6; P_t_<0.05). With the exception of *Amblyomma cajennense*, the other six species of ticks have significant lower level of heteroplasmy (15.8±13.6 sites per tick, n = 1; P_t_<0.05) than both the human lice and humans, reconfirming the view that mt genome organization has no direct influence on the level of heteroplasmy.

In conclusion, our results showed substantial variation in heteroplasmy level in mt genomes among human lice from different countries and among different species of ticks. Thus, we can reject the possible link between the elevated level of heteroplasmy and the fragmented mt genome organization in the human lice. Many questions, however, remain to be answered. Why does *cox1* have more heteroplasmy than other genes in human lice (but not in ticks)? Why do human lice from Ethiopia have higher levels of heteroplasmy than those from China and France? Why does *Amblyomma cajennense* have more heteroplasmy than other species of ticks? Are there any genomic, biochemical or life history factors that may contribute to the level of heteroplasmy? And more importantly, what does the varying level of heteroplasy mean to the fitness and evolution of individuals, populations and species? With the data generated in the current study, we cannot answer these questions yet. Both lice and ticks are ectoparasites of vertebrates. Parasitic lifestyle, however, does not appear to have any role in the variation of heteroplasmy level as there is clear difference in heteroplasmy level between lice and ticks and within each of them although all of them have the parasitic lifestyle. Recombination between mt minichromosomes observed in human lice does not appear to contribute much to heteroplasmy variation either as heteroplasmic sites are not limited to recombinant regions but spread across minichromosomes. More studies are needed to look into the issue of heteroplasmy in eukaryotes with deep sequencing technologies and to address the questions raised above in the current study.

## Supporting Information

Table S1 **Conserved and specific primers used in this study.**
(DOC)

Table S2 **Heteroplasmic sites in mitochondrial tRNA genes of human body lice and human head lice.**
(DOC)

Table S3 **Shared heteroplasmic sites in human lice.**
(DOC)

Table S4 **Heteroplasmic sites in mitochondrial tRNA genes of ticks.**
(DOC)

Table S5 **P_f_ and Q_f_ values for heteroplasmic sites at different sequence-read coverage (minimum variant 1.5%, sequencing-error rate 0.5%, π0 = 0.05).**
(DOC)

Table S6 **Putative amino acid changes caused by heteroplasmy in the 12 human lice and 7 ticks.** AA stands for amino acid. “+” stands for positive charge. “−” stands for negative charge.
(DOC)
