# Correction: Heteroplasmy in the Mitochondrial Genomes of Human Lice and Ticks Revealed by High Throughput Sequencing

**DOI:** 10.1371/journal.pone.0123492

**Published:** 2015-04-02

**Authors:** The PLOS ONE Staff

S4 Table incorrectly appears as a duplicate of S5 Table. The authors have provided a corrected version of S4 Table below.

## Supporting Information

S4 Table Heteroplasmic sites in mitochondrial tRNA genes of ticks. (DOC)
