# Supplementary material for: Heteroplasmy in the Mitochondrial Genomes of Human Lice and Ticks Revealed by High Throughput Sequencing
Source: PLoS One. 2013 Sep 13;8(9):e73329. doi: 10.1371/journal.pone.0073329 (PMC3772822; doi:10.1371/journal.pone.0073329)
Supplement: Table S1 — Conserved and specific primers used in this study. (DOC) [file pone.0073329.s001.doc]

**Table S1. Conserved and specific primers used in this study**

| Primer | Sequence 5’- 3’ | Target genes | Notes |
| --- | --- | --- | --- |
| **C1-J-1718** | GGAGGATTTGGAAATTGATTAGTTCC | ***cox1*** | Simon *et al*. (1994) |
| **C1-N-2329** | ACTGTAAATATATGATGAGCTCA | ***cox1*** | Simon *et al*. (1994) |
| SR-J-14199 | TACTATGTTACGACTTAT | ***rrnS*** | **Kambhampati & Smith (1995)** |
| SR-N-14594 | AAACTAGGATTAGATACCC | ***rrnS*** | **Kambhampati & Smith (1995)** |
| tickCOBF | CCYTGAGGNCAAATATCWTTYTGAGG | ***cytb*** | **Consensus of hard tick sequences** |
| tickCOBR | GAATATGRGGKGGDGTAATTATAGG | ***cytb*** | **Consensus of hard tick sequences** |
| tick12SF | CCATTAAAGAGCAAATTCCTCTG | ***rrnS*** | **Consensus of hard tick sequences** |
| tick12SR | CAGAGGAATTTGCTCTTTAATGG | ***rrnS*** | **Consensus of hard tick sequences** |
| Haz12SF | AAATCTCTATAAAGAAATGTAATTCAC | ***rrnS*** | For *Haemaphysalis formosensis* |
| HazCOX1F | CATCAAATTTATCTCACTATGGCCC | ***cox1*** | For *Haemaphysalis formosensis* |
| HazCOX1R | TGAGAAATTATTCCAAATCCGGGT | ***cox1*** | For *Haemaphysalis formosensis* |
| Hap12SR | TAAAGTTATGGCGGTATTTCAAGC | ***rrnS*** | For *Haemaphysalis parva* |
| HapCOX1F | ATTAGATCTTCCCTAATTGAAAGAGGAG | ***cox1*** | For *Haemaphysalis parva* |
| HapCOX1R | TTTGTGAGATTATACCAAATCCAGG | ***cox1*** | For *Haemaphysalis parva* |
| BomiCOX1F | TTATCATCAAATTTATCTCATTATGGGC | ***cox1*** | For *Rhipicephalus microplus* |
| BomiCOX1R | CTGTTAATAGTATGGTAATAGCACCTG | ***cox1*** | For *Rhipicephalus microplus* |
| Bomi12SF | CTCTTAAAAGAAATGTAATTCACTTCATTC | ***rrnS*** | For *Rhipicephalus microplus* |
| Bomi12SR | TTTCAGAGGAATTTGCTCTTTAATGG | ***rrnS*** | For *Rhipicephalus microplus* |
| BomiCOBF | TTCAGTTGACAACAATACTTTAATTCG | ***cytb*** | For *Rhipicephalus microplus* |
| BomiCOBR | TTTCTGCATCTATAAATTTGTAAGGG | ***cytb*** | For *Rhipicephalus microplus* |
| Amcaj12SF | AAAGCATTGTAATTCACTTCATCC | ***rrnS*** | For *Amblyomma cajennense* |
| AmcajCOX1F | CAATTCTTCTCTTATTGAGTCAGGG | ***cox1*** | For *Amblyomma cajennense* |
| AmcajCOX1R | AATGTGGGAAATTATACCGAATCCT | ***cox1*** | For *Amblyomma cajennense* |
| ArgCOX1F | TAATATAAGATTCTGACTCCTACCC | ***cox1*** | For *Argas sp.* |
| ArgCOX1R | AATATGGCATAGATTATTCCCAAGG | ***cox1*** | For *Argas sp.* |
| ArgCOBF | CAACTATCACCCAATGACTTTGAG | ***cytb*** | For *Argas sp.* |
| ArgCOBR | TCTAATACCAGTTAGGTCTTTAAAGGAG | ***cytb*** | For *Argas sp.* |
| Bog12SF | CATAGTGGTATACAAATTGAATTGAC | ***rrnS*** | For *Rhipicephalus geigyi* |
| BogCOX1F | TATCATCAAACTTATCTCATTATGGCCC | ***cox1*** | For *Rhipicephalus geigyi* |
| BogCOX1R | CTGTCAATAATATTGTAATAGCACCTGC | ***cox1*** | For *Rhipicephalus geigyi* |
| BogCOBF | CAACCGTAATTACAAACTTAATTTCAGC | ***cytb*** | For *Rhipicephalus geigyi* |
| BogCOBR | TTTAAATAGCATAATAACTATAGAAACTCC | ***cytb*** | For *Rhipicephalus geigyi* |
| OtmCOX1F | TTCCAACATTTCCCATTCTGGAATATCTG | ***cox1*** | For *Otobius megnini* |
| OtmCOX1R | TAATTGCTCCAGCTAATACTGGCAGAG | ***cox1*** | For *Otobius megnini* |
| OtmCOBF | ATTCTCAGTTGATAATCCAACTCTAACTCG | ***cytb*** | For *Otobius megnini* |
| OtmCOBR | GATATGGATAAATAAAGATGACTGGAAGG | ***cytb*** | For *Otobius megnini* |
