# Supplementary material for: Heteroplasmy in the Mitochondrial Genomes of Human Lice and Ticks Revealed by High Throughput Sequencing
Source: PLoS One. 2013 Sep 13;8(9):e73329. doi: 10.1371/journal.pone.0073329 (PMC3772822; doi:10.1371/journal.pone.0073329)
Supplement: Table S2 — Heteroplasmic sites in mitochondrial tRNA genes of human body lice and human head lice. (DOC) [file pone.0073329.s002.doc]

**Table S2.** Heteroplasmic sites in mitochondrial tRNA genes of human body lice and human head lice

| tRNA genes | *A* | *Q* | *N* | *E* | *D* | *H* | *T* | *L_1_* | *L_2_* | *C* | *M* | *F* | *G* | *P* | *I* | *R* | *Y* | *S_1_* | *V* | *K* | *S_2_* | *W* | Total |
| --- | --- | --- | --- | --- | --- | --- | --- | --- | --- | --- | --- | --- | --- | --- | --- | --- | --- | --- | --- | --- | --- | --- | --- |
| B2470B1 | 0 | 0 | 0 | 0 | 0 | 0 | 0 | 1 | 1 | 0 | 0 | 2 | 0 | 0 | 0 | 0 | 0 | 0 | 0 | 0 | 0 | 0 | 4 |
| B2471H5 | 0 | 1 | 0 | 0 | 0 | 0 | 0 | 1 | 1 | 0 | 0 | 1 | 1 | 0 | 0 | 4 | 0 | 1 | 0 | 0 | 0 | 0 | 10 |
| B2557B | 0 | 4 | 0 | 0 | 0 | 0 | 0 | 1 | 1 | 0 | 0 | 2 | 1 | 0 | 1 | 1 | 0 | 3 | 0 | 0 | 0 | 0 | 14 |
| B2557H | 0 | 2 | 0 | 0 | 0 | 0 | 0 | 1 | 1 | 0 | 0 | 1 | 1 | 0 | 0 | 1 | 0 | 2 | 0 | 0 | 0 | 0 | 9 |
| B2558B | 0 | 1 | 0 | 0 | 0 | 0 | 0 | 1 | 1 | 0 | 0 | 2 | 0 | 0 | 0 | 1 | 0 | 3 | 0 | 0 | 0 | 0 | 9 |
| B2558H | 0 | 2 | 0 | 0 | 0 | 0 | 0 | 1 | 1 | 0 | 0 | 2 | 0 | 0 | 0 | 1 | 0 | 3 | 0 | 0 | 0 | 0 | 10 |
| B2516B2 | 0 | 2 | 1 | 0 | 0 | 0 | 0 | 1 | 1 | 0 | 0 | 0 | 0 | 0 | 0 | 1 | 0 | 0 | 0 | 0 | 0 | 0 | 6 |
| B2516H2 | 0 | 0 | 0 | 0 | 0 | 0 | 0 | 1 | 1 | 0 | 0 | 0 | 0 | 0 | 0 | 0 | 0 | 1 | 0 | 0 | 0 | 0 | 3 |
| B2560B | 0 | 0 | 0 | 0 | 0 | 0 | 0 | 1 | 1 | 0 | 0 | 0 | 0 | 0 | 1 | 1 | 0 | 2 | 0 | 0 | 0 | 0 | 6 |
| B2560H | 0 | 0 | 0 | 0 | 1 | 0 | 1 | 1 | 1 | 0 | 0 | 0 | 1 | 0 | 0 | 2 | 0 | 1 | 0 | 0 | 0 | 0 | 8 |
| B2563B2 | 0 | 3 | 0 | 0 | 0 | 0 | 0 | 1 | 1 | 0 | 0 | 0 | 0 | 0 | 0 | 1 | 0 | 0 | 0 | 0 | 0 | 0 | 6 |
| B2563H | 0 | 2 | 0 | 0 | 0 | 0 | 0 | 1 | 1 | 0 | 1 | 3 | 1 | 0 | 0 | 2 | 0 | 3 | 0 | 0 | 0 | 0 | 14 |

*Note: Heteroplasmy was called at minimum variant frequency >1.5%, false positive rate P_f_ < 1% and false discovery rate Q_f_ < 0.1%, excluding those at sequencing error hotpots. There are two major types of sequencing error hotspots for Illumina Hiseq platform according to Nakamura et al. (2011) and Minoche et al. (2011). Type 1: after four or more identical base calls; Type 2: two adjacent SNPs;
