# Supplementary material for: Heteroplasmy in the Mitochondrial Genomes of Human Lice and Ticks Revealed by High Throughput Sequencing
Source: PLoS One. 2013 Sep 13;8(9):e73329. doi: 10.1371/journal.pone.0073329 (PMC3772822; doi:10.1371/journal.pone.0073329)
Supplement: Table S3 — Shared heteroplasmic sites in human lice. (DOC) [file pone.0073329.s003.doc]

**Table S3.** Shared heteroplasmic sites in human lice

|  | B2470B1 | B2471H5 | B2557B | B2557H | B2558B | B2558H | B2516B2 | B2516H2 | B2560B | B2560H2 | B2563B2 | B2563H |
| --- | --- | --- | --- | --- | --- | --- | --- | --- | --- | --- | --- | --- |
| *atp6* | 1 | 0 | 2 | 0 | 1 | 1 | 0 | 0 | 0 | 0 | 0 | 2 |
| *cox1* | 5 | 7 | 3 | 2 | 1 | 0 | 48 | 34 | 3 | 35 | 53 | 2 |
| *cox2* | 0 | 0 | 0 | 0 | 0 | 0 | 1 | 0 | 1 | 0 | 0 | 0 |
| *cox3* | 3 | 0 | 3 | 3 | 3 | 3 | 0 | 0 | 0 | 0 | 0 | 3 |
| *nad3* | 0 | 1 | 1 | 0 | 0 | 0 | 0 | 0 | 0 | 0 | 0 | 1 |
| *nad4* | 0 | 0 | 0 | 1 | 0 | 1 | 1 | 0 | 0 | 0 | 0 | 1 |
| *nad5* | 1 | 4 | 5 | 5 | 2 | 5 | 2 | 0 | 2 | 0 | 4 | 0 |
| *nad6* | 0 | 0 | 0 | 1 | 0 | 0 | 1 | 0 | 1 | 0 | 1 | 0 |
| *rrnL* | 0 | 0 | 2 | 3 | 0 | 0 | 1 | 0 | 2 | 0 | 1 | 0 |
| *rrnS* | 0 | 0 | 1 | 3 | 2 | 0 | 0 | 0 | 0 | 0 | 0 | 0 |
| *trnC* | 0 | 0 | 0 | 0 | 0 | 0 | 0 | 0 | 0 | 0 | 0 | 0 |
| *trnF* | 2 | 0 | 2 | 2 | 2 | 2 | 0 | 0 | 0 | 0 | 0 | 1 |
| *trnG* | 0 | 1 | 0 | 1 | 0 | 0 | 0 | 0 | 0 | 0 | 0 | 0 |
| *trnI* | 0 | 0 | 1 | 0 | 0 | 0 | 0 | 0 | 1 | 0 | 0 | 0 |
| *trnL_1_* | 1 | 1 | 1 | 1 | 1 | 1 | 1 | 1 | 1 | 1 | 1 | 1 |
| *trnL_2_* | 1 | 1 | 1 | 1 | 1 | 1 | 1 | 1 | 1 | 1 | 1 | 1 |
| *trnQ* | 1 | 1 | 2 | 2 | 1 | 2 | 2 | 0 | 0 | 0 | 2 | 1 |
| *trnR* | 0 | 2 | 1 | 0 | 1 | 1 | 1 | 0 | 1 | 0 | 0 | 2 |
| *trnS_1_* | 0 | 1 | 2 | 2 | 3 | 2 | 0 | 1 | 2 | 1 | 0 | 2 |
| Total | 15 | 19 | 27 | 27 | 18 | 19 | 59 | 37 | 15 | 38 | 63 | 17 |
