# Supplementary material for: Heteroplasmy in the Mitochondrial Genomes of Human Lice and Ticks Revealed by High Throughput Sequencing
Source: PLoS One. 2013 Sep 13;8(9):e73329. doi: 10.1371/journal.pone.0073329 (PMC3772822; doi:10.1371/journal.pone.0073329)
Supplement: Table S4 — Heteroplasmic sites in mitochondrial tRNA genes of ticks. (DOC) [file pone.0073329.s004.doc]

**Table S5.** P_f_ and Q_f_ values for heteroplasmic sites at different sequence-read coverage (minimum variant 1.5%, sequencing-error rate 0.5%, π_0_ = 0.05)

| Coverage | P_f_ | Q_f_ |
| --- | --- | --- |
| 23 | 0.477031 | 0.05025621 |
| 132 | 0.115718 | 0.006171841 |
| 218 | 0.0522015 | 0.002872436 |
| 279 | 0.0311552 | 0.001797212 |
| 328 | 0.0209281 | 0.001237155 |
| 423 | 0.0099482 | 0.0006331697 |
| 512 | 0.00506857 | 0.000338389 |
| 733 | 0.00100388 | 8.407053e-05 |
| 960 | 0.000199598 | 2.165411e-05 |
| 5013 | 2.8177e-16 | 3.164774e-16 |
| 12223 | 6.46736e-37 | 8.575744e-36 |
