# Supplementary material for: Heteroplasmy in the Mitochondrial Genomes of Human Lice and Ticks Revealed by High Throughput Sequencing
Source: PLoS One. 2013 Sep 13;8(9):e73329. doi: 10.1371/journal.pone.0073329 (PMC3772822; doi:10.1371/journal.pone.0073329)
Supplement: Table S6 — Putative amino acid changes caused by heteroplasmy in the 12 human lice and 7 ticks. AA stands for amino acid. “+” stands for positive charge. “−” stands for negative charge. (DOC) [file pone.0073329.s006.doc]

**Table S6.** Putative amino acid changes caused by heteroplasmy in the 12 human lice and 7 ticks. AA stands for amino acid. “+” stands for positive charge. “-” stands for negative charge.

| Human lice AA change | | Ticks AA change | |
| --- | --- | --- | --- |
| Hydrophilic^-^ → Hydrophilic^-^ | 3 | Hydrophilic^-^ → Hydrophilic^-^ | 0 |
| Hydrophilic^-^ → Hydrophilic^+^ | 0 | Hydrophilic^-^ → Hydrophilic^+^ | 2 |
| Hydrophilic^-^ → Hydrophilic (non-charge) | 2 | Hydrophilic^-^ → Hydrophilic (non-charge) | 0 |
| Hydrophilic^+^ → Hydrophilic^-^ | 2 | Hydrophilic^+^ → Hydrophilic^-^ | 0 |
| Hydrophilic^+^ → Hydrophilic (non-charge) | 5 | Hydrophilic^+^ → Hydrophilic (non-charge) | 1 |
| Hydrophilic (non-charge) → Hydrophilic^-^ | 2 | Hydrophilic (non-charge) → Hydrophilic^-^ | 2 |
| Hydrophilic (non-charge) → Hydrophilic^+^ | 14 | Hydrophilic (non-charge) → Hydrophilic^+^ | 2 |
| Hydrophobic → Hydrophilic^+^ | 0 | Hydrophobic → Hydrophilic^+^ | 5 |
| Hydrophilic (non-charge)→ Hydrophilic (non-charge) | 19 | Hydrophilic (non-charge) → Hydrophilic (non-charge) | 5 |
| Hydrophilic (non-charge) → Hydrophobic | 20 | Hydrophilic → Hydrophobic | 11 |
| Hydrophobic → Hydrophilic (non-charge) | 26 | Hydrophobic → Hydrophilic (non-charge) | 10 |
| Hydrophobic → Hydrophobic | 29 | Hydrophobic → Hydrophobic | 25 |
